# On Recent Improvements in Surgery

**Published:** 1842-12-10

**Authors:** 


### BIBLIOGRAPHICAL NOTICES.

*On Recent Improvements in Surgery. An Introductory Lecture to the course on the Principles and Practice of Surgery, in Jefferson Medical College of Philadelphia.* Delivered November 3, 1842. By THOMAS D. MÜTTER, M. D. Published by the class. Philadelphia: 8vo. pp. 52.

This is a rapid and tolerably complete *aperçu* of the progress of Surgical Science for the past few years. A laudable industry in the collection of material, and in general a just appreciation in judgment are shown. We extract the conclusion.

"In this rapid and hasty sketch of a portion of what has occupied the time and talents of our brethren for some years past, it must have been obvious to you that American surgeons have been no laggards in the mighty race for professional fame. That they as well as their collaborators in Europe, have been steadily engaged in adding each a stone to the pyramid of modern surgery! The flame which burns so brilliantly abroad has thrown its rays across the wide Atlantic, and soon its genial warmth will be felt from one extremity of our country to the other. Shall it be permitted to subside? Will you who are destined to be the pillars upon which the medical science of this country is to rest, fail to add fuel to this flame? Will you by slothful indulgence, wasteful sensual gratification, ignoble and puny contentedness which readily *receives* but never *gives*, let pass this golden era? Will you not rather 'gird up your loins' to the toil—and by your diligence, morality, and laudable ambition, wreathe a new chaplet of glory for the land of liberty and equal rights? Show to the world that if in politics, religious tolerance, and social virtue, America once stood and will stand again, the foremost of nations, she may also boast of her medical science.

"Recollect that you are destined to sustain the honours and prolong the glories of a science that has long been dignified and adorned by the profound attainments, the elevated integrity, the high bred courtesy of a Hunter, a Cooper, a Bell, a Paré, a Dupuytren, and a Physic!—What an inspiring example! What an animating incentive! How proud your obligation! With those lights to guide you in the path to excellence, hasten to prove yourselves worthy of receiving the bright mantle of fame with which a grateful world has always been ready to clothe him who with tenderness and love ministers to the pangs and sorrows of his fellow man.

"There are many among you who are discouraged from entering with ardor upon the pursuit of the profession, from the supposition that nothing, or next to nothing remains for them to discover. Let no such idea take possession of your minds; ours, as I have already told you, is a progressive science, and far, very far, from perfection. Mirabeau has justly remarked 'that to suppose every thing in any science to be discovered, is like taking the horizon for the limits of our earth!' Fully impressed with this fact, let me urge upon you constant, patient, unprejudiced investigation. The harvest is rich, and he who boldly thrusts in his sickle will assuredly reap an abundant reward. It is true that 'generation after generation will probably pass away ere a final victory over the chaos and confusion which still reigns over us can be accomplished, but the day will come when our descendants, like the discoverers of old at the pillars of Hercules, may pause and say—'

'Hic tandem stetimus, nobis ubi deficit orbis!'

Dwell not then upon what has been done but what remains to do.

"Another influence well calculated to dampen you energies is the success of quackery. The dark clouds of ignorance, and error, and presumption do indeed gather more and more around and above us, and often the true votaries of our noble art are bowed down with despair. It comes not within my province to discuss the causes of this increase of the evil, but there is surely none more efficient than the facility with which a medical degree is usually obtained, and consequently the little value set upon its possession by the community at large. I trust, however, that a better state of things will sooner or later arise, and in the mean time should any among you be disheartened, let him not look to the boasted success of the lying and impudent quack, but to the brilliant examples of wisdom, and intellect, and honesty with which our profession is replete. In whatever direction we turn, the trophies of these great men are before us, and pointing to them, gentlemen, I would appeal to you in the language of Demosthenes when pleading in behalf of the Rhodians, he invoked the memory of the illustrious dead and pointed to the monuments of their valour to rouse their sons to the same noble achievements; 'think,' says he, 'that your ancestors erected these trophies not that the view might barely strike you with admiration, but that *you* might imitate the virtues of the men who raised them!' Allow not then the temptings of the demon of avarice to lead you astray—the gains of the mercenary and hard hearted empiric, though often alluring to the poor and diligent, but honest and honourable votary of his art, can in the end be productive of no comfort, no satisfaction. I envy not the man whose highest pinnacle of professional eminence is based upon money, whose loftiest aspirations are bounded by an horizon of gold. Alas! he knows not the charm that envelopes and hallows the charitable act; he feels not the genial warmth which springs from the poor man's blessing; he cares not for the tears of the orphan and widow; he thinks of and values only, the means by which he may heap up the poor, fleeting, perishable wealth of this world, and with every generous feeling callous, with every kindly sympathy locked up in his frigid heart,he moves in the crowd not as a dispenser of health and comfort to the sick and needy; not as the cherished friend of every homestead; not as the harbinger at whose approach the pangs of death, even, are subdued; but rather as the harpy, who preys upon the very vitals of his patient. Oh then, gentlemen, cherish the kindly feeling of our nature—degrade not by sordid motives, that magnificent vocation, that noble and admirable mission which shrinks from no devotion and distributes it success impartially to the crowned head and to the poor beggar —and in conclusion let me remind you in the glowing stanzas of a native bard, that

'Art is long and time is fleeting,
And our hearts though stout and brave,
Still like muffled drums are beating
Funeral marches to the grave!

Let us then be up and doing,
With a heart for any fate;
Still achieving, still pursuing,
Learn to labour and to wait.'"

LONGFELLOW.

